# HIV prevention metrics: lessons to be learned from contraception

**DOI:** 10.1002/jia2.25958

**Published:** 2022-08-05

**Authors:** Maria Pyra, Renee Heffron, Jessica E. Haberer, James Kiarie

**Affiliations:** ^1^ University of Chicago Chicago Illinois USA; ^2^ University of Washington Seattle Washington USA; ^3^ Massachusetts General Hospital Boston Massachusetts USA; ^4^ World Health Organization Geneva Switzerland

**Keywords:** HIV care continuum, HIV prevention, key and vulnerable populations, PrEP, adherence, retention

## Abstract

**Introduction:**

As the range of effective HIV prevention options, including multiple biomedical tools, increases, there are many challenges to measuring HIV prevention efforts. In part, there is the challenge of varying prevention needs, between individuals as well as within individuals over time. The field of contraception faces many similar challenges, such as the range of prevention methods and changing contraceptive needs, and has developed many metrics for assessing contraceptive use at the program level, using frameworks that move beyond the HIV prevention cascade. We explore these similarities and differences between these two prevention fields and then discuss how each of these contraceptive metrics could be adapted to assessing HIV prevention.

**Discussion:**

We examined measures of initiation, coverage and persistence. Among measures of initiation, HIV Prevention–Post Testing would be a useful corollary to Contraceptive Use–Post Partum for a subset of the population. As a measure of coverage, both Net Prevention Coverage and HIV Protection Index (modelled off the Contraception Protection Index) may be useful. Finally, as a measure of persistence, Person‐Years of HIV Protection could be adapted from Couple‐Years Protection. As in contraception, most programs will not reach 100% on HIV prevention metrics but these metrics are highly useful for making comparisons.

**Conclusions:**

While we may not be able to perfectly capture the true population of who would benefit from HIV prevention, by building off the work of the contraceptive field to use and refine these metrics, we can assess and compare HIV prevention over time and across programs. Furthermore, these metrics can help us reach global targets, such as the 2025 UNAIDS Goals, and reduce HIV incidence.

## 1 INTRODUCTION

As highly effective HIV prevention methods—including treatment as prevention, HIV pre‐exposure prophylaxis (PrEP), condoms and male circumcision—have been available for more than a decade now, there are increasing challenges to measuring HIV prevention efforts. Some programs have focused strongly on PrEP uptake and refills, whereas others have taken a more holistic approach, including sexual behaviours and condom use. While the ultimate measure of HIV prevention is reduction in HIV incidence, it is a costly outcome to measure. By developing better measures of HIV prevention use, we can assess and improve prevention programs to minimize new cases. Many metrics have focused on oral PrEP, and HIV prevention cascades have often been constructed to mirror HIV care cascades [1, 2, 3, 4, 5, 6, 7, 8, 9, 10]. While these cascades, which track the number of individuals at successive steps of engagement, may be useful for understanding knowledge or motivation and identifying implementation strategies [2, 9, 11], the linear nature of HIV care cascades (diagnosis, treatment and viral suppression) does not translate well to PrEP use, as has been previously noted [12]. Specifically, PrEP is often used episodically or cyclically, driven by intermittent need for protection during periods of potential HIV exposure [13].

Contraceptives have been previously explored in various contexts as a comparator to PrEP [14, 15]. People use contraception and HIV prevention according to changing life situations as well as individual values and preferences; both are associated with challenges in measuring the underlying risk; and now, both have multiple options and formulations approved for use [14, 15, 16, 17]. As HIV prevention and contraception are part of the same sexual and reproductive health umbrella (as highlighted in the UNAIDS report on HIV targets for 2025 [18]), each can be useful in informing the successful delivery of the other. In this article, we continue efforts to utilize contraception experience and methods to examine metrics that could be adapted for HIV prevention with the goal of highlighting pragmatic measures to be used at the clinic or system level to evaluate, compare and improve the delivery of HIV prevention, including biomedical prevention.

Before exploring specific metrics, there are important themes around contraception and HIV prevention that are worth considering. First, the contraception field encourages incorporating multiple methods in metrics. For instance, contraception metrics often account for biomedical methods, as well as abstinence, abortion, infertility and partner sterilization. The corollary in HIV prevention could be to include undetectable equals untransmittable (U = U), consistent condom use and male circumcision, in addition to PrEP, to define a suite of HIV prevention methods, as seen in the UNAIDS 2025 target report [18].

Second, contraceptive options are often grouped by their effectiveness. Therefore, HIV prevention options could be grouped with daily oral PrEP, U = U and condoms (in partnerships between men) considered highly efficacious, while male circumcision less so [15, 19, 20, 21, 22]. As multiple PrEP products become available, further distinctions and metrics that capture all the different options and their effectiveness will be all the more important. Unlike contraception, HIV prevention must also consider non‐sexual routes of transmission, such as drug use, and relevant prevention methods, such as using clean needles.

Third, measurements of contraception and HIV prevention struggle with quantifying denominators, that is the number who are at risk of becoming pregnant or who would benefit from HIV prevention, respectively. Contraception metrics often first restrict by sex and age—because these greatly impact fertility—and then use proxies, such as marital status, sexual activity or post‐partum status to further refine the number with potential to become pregnant. For instance, the term unmet need often uses “married women” as the population at risk, even though this clearly does not capture the true population who could have an unintended pregnancy; the term is being replaced by more appropriate language, such as “sexually active” [23]. However, it may not be possible to know how many women (or their partners) suffer from infertility or are having mistimed conception. Likewise, HIV prevention needs are expected to change over time and potential exposures to HIV are difficult to measure. For instance, relationships may end or become monogamous; partners may become virally suppressed. Yet, this approach suggests that imperfect denominators can still be useful for real‐world comparisons. For this article, we use the term “would benefit from” as a place holder for identifying the denominator for HIV prevention metric; once individuals are using effective prevention of any form, they would no longer be in this category. Many researchers and healthcare practitioners have highlighted the need to move away from “at risk” language and to broaden access and aware of HIV prevention methods, to decrease stigma and increase uptake [24, 25]. We also highlight ways in which the denominator could be adapted based on the availability of data and specific populations in a clinic or program.

In addition, for almost all measures of contraceptive use, there is not the expectation to reach 100% [26]. As the true population that would benefit from HIV prevention is almost impossible to define, appropriate expectations are important for HIV prevention as well. Most HIV prevention metrics will not reach 100% but can still be useful in making comparisons between programs, between sub‐populations and over time. Realistic expectations are important to avoid inappropriate dismissal of prevention options that provide meaningful, yet imperfect, benefit.

Finally, contraceptive metrics are also used to provide the component data to assess progress towards larger, global targets [27, 28]. For example, the Family Planning 2020 initiative clearly laid out what metric would be used (or need to be developed) for each step of their logic model [29]. Likewise, we anticipate that these specific HIV prevention metrics can improve program success and help achieve larger, global goals, such as the 2025 targets [18].

## 2 DISCUSSION

A range of contraception metrics is presented in Table 1 that spans various adherence concepts. Some of the oldest and best known, such as unmet need, are very informative, but also highly burdensome to collect and calculate [23]. Newer measures have also been proposed to address limitations of earlier measures, which can advance the work of developing improved HIV prevention metrics. Interestingly, while PrEP research has focused in large part on adherence—whether through self‐report, pill counting, electronic monitoring or a variety of biomarkers—this same emphasis is lacking in contraception; therefore, most contraception measures presented reflect cross‐sectional analysis. Indeed, both HIV PrEP and contraception metrics often focus on initiation, which is more straightforward to quantify; understanding seasonal or prevention‐effective use of PrEP (i.e. use that is aligned with needed benefit) is an ongoing effort [13].

**Table 1:** Summary of contraception metrics and suggested HIV prevention adaptations

Contraceptive metric	Definition	Adapted HIV prevention metric	Definition of HIV prevention adaptation	Notes on use/limitations
Initiation				
Contraceptive care‐post‐partum (CCP) [26]	# reproductive age women with a live birth *provided* an effective method within 60 days / # reproductive age women with a live birth	HIV prevention‐post testing (HPP)^a^	# with a negative HIV test *provided* effective HIV prevention within 2 weeks / # with negative HIV test	Additional factors could be added to better define potential benefit among specific clinic populations. The time window could vary and may be more easily assessed as same day.
Contraceptive care–effective methods (CCE) [26]	# reproductive age women at risk for unintended pregnancy *provided* most or moderately effective method / # of reproductive age women at risk for unintended pregnancy	HIV prevention—effective methods (HPE)	# who would benefit *provided* effective HIV prevention / # who would benefit	For example, “would benefit” could include all sexually active patients or all people who inject drugs. Effective HIV prevention could be defined as on PrEP, using U = U, not sharing needles and/or consistent condom use. The metric is limited by inaccuracy in risk assessment.
Coverage				
	–	Net Prevention Coverage (NPC)^a^ [34]	((# no anal intercourse with casual partners of any HIV status) + (# consistent condom use with casual partners of any HIV status) + (# U = U with casual partners living with HIV) + (# using PrEP)) / # HIV‐negative respondents	NPC is specifically tailored to MSM, where casual sex has been identified as the major factor in HIV acquisition, although it can be adapted to other populations. It is limited by accuracy in knowledge of who would benefit.
Contraception Protection Index (CPI) [33]	Σ (Effectiveness of method_1_ x % of women using method_1_) + (Effectiveness of method_n_ x % of women using method_n_)	HIV Protection Index (HPI)^a^	Σ (effectiveness of method_1_ x % of people using method_1_) + (effectiveness of method_n_ x % of people using method_n_)	HPI would require data on actual use (vs. perfect use) effectiveness of HIV prevention methods, which could be estimated from robust clinical data.
Unmet need [23, 30]	# married women not using contraception + (# married women pregnant or immediately postpartum) + (# married women pregnant or postpartum wanting to delay or not have more children) + (# married women able to have children and wanting to delay or not have more children)	Unmet protection	# would benefit and without effective HIV prevention	For example, “would benefit” could include all sexually active patients or all people who inject drugs. Effective HIV prevention could be defined as on PrEP, using U = U, not sharing needles and/or consistent condom use. This metric is limited by inaccuracies in risk and use assumptions or assessments.
Modern contraceptive prevalence rate (MCPR) [31]	# reproductive age women using condoms, pills, implants, injectables, IUDs or sterilization/# reproductive age women	Modern prevention prevalence rate (MPPR)	# would benefit and on effective HIV prevention/# would benefit	For contraception, who would benefit is defined slightly differently; for HIV prevention, would be equivalent to CCE
Persistence				
Couple‐Years Protection (CYP) [38]	Σ (# doses of method_1_ x duration of dose_1_) + (# doses of method_n_ x duration of dose_n_)	Person‐Years HIV Protection (PYHP)^a^	Σ (# doses of method_1_ x duration of dose_1_) + (# doses of method_n_ x duration of dose_n_)	This metric does not include a denominator. It could also be considered a measure of coverage. It should not be used to compare year to year, as high protection in year 1 may cover future years; instead, it can be annualized over duration [38]. It does not comment on effectiveness of the method.
Contraceptive continuation rates (CCR) [39]	(1–(# women discontinuing during interval_1_/# women using at start of interval_1_)) x (1–(# women discontinuing during interval_n_/# women using at start of interval_n_))	HIV prevention continuation rates (HPCR)	(1–(#Method users stopping during interval_1_/# Method users at start of interval_1_)) x (1–(#Method users stopping during interval_n_/# Method users at start of interval_n_))	Discontinuation must be defined for each method of HIV prevention. Use of CCR may be limited by the need for intensive resources to measure behaviours and associated inaccuracies.

^a^ Recommended metrics for HIV prevention.

### 2.1 Initiation and coverage

The measures described in this section consider the use of a prevention method at a particular time‐point. We consider measures of starting a prevention method to describe initiation and those that indicate prevalence to describe coverage.

We highlight two measures of contraception initiation. The first relates to an important reproductive event, with contraceptive care‐post‐partum (CCP) defined as the proportion of women who gave birth and were provided an effective method within 60 days [26]. The second relates to women at risk for an unintended pregnancy, contraceptive care‐effective methods (CCE), which is defined as the proportion of women at risk for unintended pregnancy and provided a mostly or moderately effective method [26]. In many ways, this metric echoes the concept of unmet need and faces similar challenges of defining who would benefit from prevention. An HIV prevention corollary derived from both CCP and CCE could be sexually active individuals who have a sexually transmitted infection and/or HIV test (as markers of recent exposure, similar to post‐partum status) and are using effective HIV prevention within a reasonable time frame (e.g. 2 weeks post‐testing or same‐day). Community prevalence rates of HIV and partnership types could also be an important component of defining who would benefit from PrEP, as the chance of acquiring HIV is not uniform among sexually active people. Definitions should take into account the availability of programmatic data—for instance, ability to follow‐up a patient if prevention is not provided same‐day or to access testing results.

There are three measures of contraceptive coverage: unmet need, modern contraceptive prevalence rate (MCPR) and Contraceptive Protection Index (CPI). Conceptually, unmet need is easy to understand; it enumerates the gap in contraceptive access, among those who want to avoid pregnancy [30]. However, the actual calculation of unmet need requires long surveys to determine reproductive ability and fertility desires, including if and when a woman wants another child [23]. For HIV prevention, the corollary may be unnecessarily complicated, particularly as we can assume that all persons without HIV would want to remain uninfected. MCPR [31] uses a simpler formula to define the denominator (i.e. reproductive age women) and includes all forms of “modern” contraceptive (i.e. condoms, pills, implants, injectables, intrauterine devices [IUDs] and sterilization) users in the numerator, as a cross‐sectional measure; this excludes less effective methods, such as withdrawal, breastfeeding or calendar/rhythm methods. The trade‐off of using a simpler denominator is ignoring important heterogeneity—the chances of pregnancy are not the same for all women of reproductive age; this same process can be applied to HIV prevention denominators. One criticism of MCPR is that it does not account for the effectiveness of different methods; condom use and sterilization are equally weighted [32]. For an HIV prevention metric, the denominator is less well‐defined and often reflects key populations, such as sex workers, young women or men who have sex with men (MSM), who may have heterogeneous benefits from HIV prevention. However, in creating an MCPR type measure, virtually all HIV biomedical preventions would be considered “modern,” making the distinction around modern methods less meaningful. We, therefore, would not recommend a corollary of MCPR for HIV prevention. Building off the effectiveness criticism of MCPR, the CPI weights each method by its real‐world effectiveness, among women using each method [33]. This recommended approach would more easily translate to the variety of HIV prevention options, particularly as more forms of PrEP become available and more data on long‐term effectiveness of treatment as prevention and PrEP are collected.

One additional measure of coverage came out of the HIV prevention field itself, Net Prevention Coverage (NPC); this measure, developed by Holt et al., accounts for the variety of “modern” HIV prevention methods, including U = U and consistent condom use, although it does not weight by the effectiveness of each method [34]. This approach requires the collection of data not typically captured in routine clinical care, such as classifying partners as casual, but also shows the usefulness and feasibility of broader HIV prevention metrics. Thus far, NPC has been used with sexually active MSM and addresses the problem of defining who would benefit by focusing only on casual partners (which is a key driver in this population); developing versions of this metrics for other populations would be a useful area of research. The use of sexual history questionnaires at the clinic level or larger behavioural surveys at the population level are potential data collection methods that could provide useful data to adapt this approach to other contexts [35, 36, 37].

### 2.2 Persistence

Measures of persistence include some indication of duration of use or at least availability. Two key measures of persistence from contraception are Couple‐Years Protection (CYP) and contraceptive continuation rates (CCR). CYP is a clinic‐level measure that could be easily adapted to PrEP [38]. It is a measure of how many doses of each contraceptive type were delivered, multiplied by the protective period of each type. For instance, a pack of oral birth control pills would protect 30 days, while an injection of depot medroxyprogesterone acetate would protect 90 days. This approach would readily translate to various types of HIV prevention (e.g. a 90‐day supply of PrEP would generally protect 90 days, while a single cabotegravir injection would protect 60 days). The larger question may be whether this metric truly captures protection of the “couple” or just the individual; we suggest reframing it for the individual (i.e. Person‐Years Protection), as the other partner(s) may have their own protection needs or already be living with HIV. While the value of CYP is clear, stakeholders need to understand that long‐acting methods can result in lower CYP values in future years, while still equalling high protection [32]; in other words, an implant that provides 5 years of protection is only counted in year 1. While CYP represents potential protection, CCR accounts for discontinuation rates by method, thereby trying to account for actual use with an end‐result similar to a demographic life table [39]. The CCR requires careful data collection to validate the discontinuation rates; these rates could vary greatly across different populations using PrEP and would be difficult to account for prevention‐effective use, which changes over time. Therefore, CCR is a less practical measure.

## 3 CONCLUSIONS

The field of contraception has been working for decades to reduce unintended pregnancies by delivering prevention methods to a population that is challenging to enumerate, in terms of assessing the actual probability of pregnancy, and has changing reproductive needs. In doing so, they have developed and refined a range of metrics to help assess programs and work towards global targets [27, 29]. Likewise, HIV prevention is working to deliver a variety of prevention methods to reduce HIV incidence, with similar measurement concerns. There are also global targets for HIV prevention—most recently, the UNAIDS 2025 target of 95% of key populations using prevention [18]. We propose adapting a variety of contraceptive metrics to guide progress to these global targets and help individual programs assess their work. We highlight the metrics that we think best fit HIV prevention—HIV Prevention–Post Testing, NPC, HIV Protection Index, and Person‐Years HIV Protection—particularly as new formulations of PrEP become available, and we encourage a focus beyond just biomedical interventions to include other prevention methods (U = U, consistent condom use, syringe exchange, etc. [18]). As in contraception, it is not possible to perfectly predict who will acquire HIV (or become pregnant), but imperfect denominators can still be informative. Meeting the 2025 targets may require even closer collaboration between contraception and HIV prevention, as combination prevention is the goal [18]. By beginning to use and refine these proposed measures, we can assess and improve delivery of HIV prevention over time. To paraphrase the statistician George Box, “All metrics are imperfect, but some are useful”; the goal of this paper is to move the HIV prevention field closer to the latter.

## COMPETING INTERESTS

The authors declare no competing interests.

## AUTHORS’ CONTRIBUTIONS

MP developed the research question. MP, RH, JEH and JK all contributed to writing and editing of the manuscript.
